# Prevalence of body weight dissatisfaction among adolescents: a systematic review

**DOI:** 10.1590/1984-0462/2023/41/2021204

**Published:** 2022-09-12

**Authors:** Mariana Contiero San Martini, Daniela de Assumpção, Marilisa Berti de Azevedo Barros, Josiemer Mattei, Antônio de Azevedo Barros

**Affiliations:** a Universidade Estadual de Campinas, Campinas, SP, Brazil.; b Harvard T. H. Chan School of Public Health, Boston, MA, United States.

**Keywords:** Body weight, Body dissatisfaction, Adolescents, Health surveys, Public health, Peso corporal, Insatisfação corporal, Adolescentes, Inquéritos epidemiológicos, Saúde pública

## Abstract

**Objective:**

To identify the prevalence of weight dissatisfaction among adolescents aged 10-19 years and stratify the analysis by sex.

**Data source:**

A literature review of cross-sectional studies among healthy adolescents was performed. The U.S. National Library of Medicine/National Institutes of Health (PubMed), Ovid^®^ (Wolters Kluwer), The Cumulative Index to Nursing and Allied Health Literature (CINAHL), and American Psychological Association (PsycINFO^®^) databases were searched between May 2019 and January 2020.

**Data synthesis:**

Initially, 3,700 records were identified, and 10 papers were obtained through other sources. After the removal of duplicates, 1,732 records were screened based on the titles and abstracts, and 126 were preselected for full-text analysis. After the application of the eligibility criteria, 34 papers were included in the present review. The studies were published between 1997 and 2020. The sample size ranged from <150 to >103,000 adolescents. The prevalence of weight dissatisfaction ranged from 18.0 to 56.6% in both sexes (10.8-82.5% among boys and 19.2-83.8% among girls).

**Conclusions:**

Based on the findings of the present systematic review, the prevalence of weight dissatisfaction is high among adolescents, especially girls. Such information can contribute to the planning of health and education programs addressing the issue of weight in adolescents.

## RESUMO

**Objetivo:**

Identificar as prevalências de insatisfação com o peso corporal em adolescentes de dez a 19 anos, segundo o sexo.

**Fontes de dados:**

Foram selecionados estudos transversais que descreviam as prevalências de insatisfação com o peso em adolescentes saudáveis, nos idiomas inglês, português e espanhol. Não foram estabelecidos limites para o ano de publicação ou país. Utilizaram-se as bases de dados *US National Library of Medicine/National Institutes of Health* (PubMed), Ovid^®^ (Wolters Kluwer), *The Cumulative Index to Nursing and Allied Health Literature* (CINAHL) e *American Psychological Association* (PsycINFO^®^), entre maio de 2019 e janeiro de 2020.

**Síntese dos dados:**

Inicialmente identificaram-se 3.700 registros e dez estudos em outras fontes de dados. Após a remoção dos estudos duplicados, foram rastreados 1.732 registros para a leitura de títulos e resumos, dos quais 126 artigos foram selecionados para avaliação do texto completo. Por fim, foi incluído nesta pesquisa o total de 34 artigos. Os estudos foram publicados entre 1997 e 2020. Os tamanhos amostrais variaram de <150 a >103.000 adolescentes. As prevalências de insatisfação com o peso variaram de 18,0 a 56,6% em ambos os sexos (10,8 a 82,5% no sexo masculino e 19,2 a 83,8% no feminino).

**Conclusões:**

Esta revisão identificou elevada prevalência de insatisfação com o peso corporal em adolescentes, superior em meninas. Tais informações podem contribuir para o planejamento de programas de saúde e educação direcionados à questão do peso em adolescentes.

## INTRODUCTION

Dissatisfaction with one’s body weight reflects the desire to modify one’s current weight.^1^ This is a common occurrence^2^ ^,^ ^3^ with differences between the sexes.^4^ Indeed, weight dissatisfaction is more prevalent in the female sex throughout life.^5^ ^,^ ^6^

Adolescent girls often want to lose weight and have a thinner body, whereas adolescent boys desire a more muscular, athletic body and often wish to gain weight.^5^ ^,^ ^6^ ^,^ ^7^ ^,^ ^8^ However, dissatisfaction with one’s weight does not necessarily imply body dissatisfaction.^9^

The prevalence of weight dissatisfaction is considered a significant public health problem by health professionals around the world.^3^ Data on dissatisfaction with body weight are important to the planning of prevention measures targeting excess weight and the promotion of weight loss or the maintenance of a healthy weight.^10^

There is currently a fear of fat,^3^ the stigmatization of obesity, and the idealization of thinness as synonymous with health^11^ or even more important than health.^12^ Weight dissatisfaction can contribute to the development of eating disorders, harmful weight control strategies, depression, and low self-esteem, interfering with the physical and emotional development of adolescents.^3^

Therefore, the aim of this study was to perform a systematic review of the literature on the prevalence of body weight dissatisfaction among adolescents aged 10-19 years.

## METHOD

This study was conducted, following the Preferred Reporting Items for Systematic Reviews and Meta-Analyses (PRISMA statement).^13^

The inclusion criteria were studies with a cross-sectional design that estimated the prevalence of weight dissatisfaction among healthy male and female adolescents between 10 and 19 years of age (based on the definition of the World Health Organization [WHO]).^14^ This age group was chosen due to its greater vulnerability. Studies published in English, Portuguese, and Spanish were selected. No restrictions were imposed regarding the year of publication or the country in which the study was developed.

We excluded studies that evaluated individuals with health problems, such as eating disorders, chronic noncommunicable diseases, Down’s syndrome, heart disease, liver disease, congenital defects, or other diseases that compromise growth and development; adolescents in treatment with corticoids; those with edema; those undergone bariatric/esthetic/sex-change surgery; pregnant girls; institutionalized individuals; and those belonging to groups/occupations overly concerned with thinness, physical appearance, and body image, such as models, actors, dancers, singers, and athletes. Articles that described the perception of weight and/or body image and/or body shape and did not evaluate weight dissatisfaction were excluded. Systematic reviews, meta-analyses, abstracts presented at conferences, comments, editorials, letters, case reports, news, theses, dissertations, and books were also excluded.

Searches were conducted in the U.S. National Library of Medicine/National Institutes of Health (PubMed), Ovid^®^ (Wolters Kluwer), The Cumulative Index to Nursing and Allied Health Literature (CINAHL), and American Psychological Association (PsycINFO^®^) databases between May 2019 and January 2020, with the following descriptors (MeSH) and respective synonyms, free terms, connectors, and filters: (Body Weight OR Ideal Body Weight OR Healthy Weight OR Weight Satisfaction OR Weight Dissatisf*) AND (Personal Satisfaction OR Dissatisfaction OR Dissatisfied OR Satisfaction OR Satisfied) AND (Adolescent OR Adolescence OR Adolescents OR High School* OR Highschool* OR Junior High OR Middle School* OR Preadolescen* OR Prepubert* OR Prepubescen* OR Preteen* OR Pubertal OR Puberty OR Pubescen* OR Secondary School* OR Teen OR Teenage* OR Teens). The full electronic search strategy of the PubMed database is presented in Board 1. Searches were also performed in Google Scholar and the reference lists of the selected articles. The researchers had several meetings with librarians for the purposes of clarification.

**Board 1:** Full electronic search strategy of the PubMed database.

(“Body Weight”[Mesh:NoExp] OR “Ideal Body Weight”[Mesh] OR body weight[tiab] OR healthy weight[tiab] OR weight satisfaction[tiab] OR weight dissatisf*[tiab]) AND (“Personal Satisfaction”[Mesh] OR dissatisfaction[tiab] OR dissatisfied[tiab] OR satisfaction[tiab] OR satisfied[tiab]) AND (“Adolescent”[Mesh] OR adolescence[tiab] OR adolescent[tiab] OR adolescents[tiab] OR high school*[tiab] OR highschool*[tiab] OR junior high[tiab] OR middle school*[tiab] OR preadolescen*[tiab] OR prepubert*[tiab] OR prepubescen*[tiab] OR preteen*[tiab] OR pubertal[tiab] OR puberty[tiab] OR pubescen*[tiab] OR secondary school*[tiab] OR teen[tiab] OR teenage*[tiab] OR teens[tiab]) AND (English[lang] OR Portuguese[lang] OR Spanish[lang]) NOT (Comment[ptyp] OR Editorial[ptyp] OR Letter[ptyp] OR Case Reports[ptyp] OR News[ptyp])

**Figure 1:**
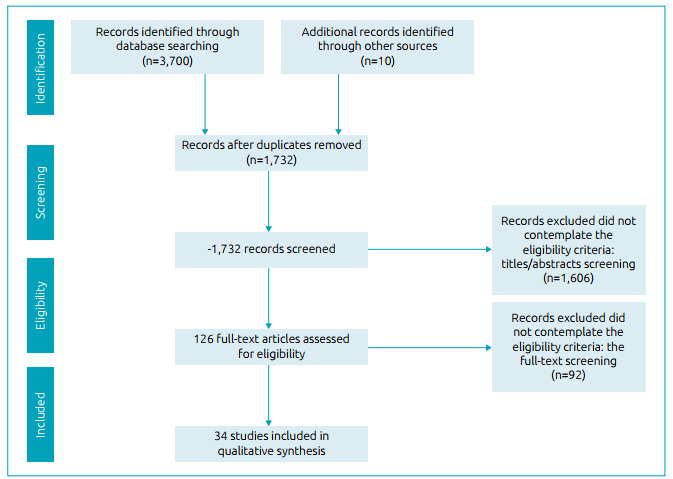
PRISMA flow diagram.^13^

The searches of the databases led to the identification of 3,700 records, and 10 studies were retrieved from other sources (Google Scholar and the reference lists of the selected articles). After the removal of 1,978 duplicates, 1,732 articles were submitted to an analysis of the titles and abstracts. A total of 1,606 articles did not meet the eligibility criteria and 126 articles were preselected. The full-text analysis led to the exclusion of another 92 articles. Thus, 34 articles met the eligibility criteria and were included in the present systematic review. A flow diagram that displays all steps of the article selection process was performed according to the PRISMA guidelines (Figure 1). The EndNote X9 software program was used for the extraction of the data.

After the exclusion of duplicate records, two reviewers preselected articles based on readings of the titles and abstracts, considering the previously established eligibility criteria. Divergences of opinion regarding the inclusion/exclusion of an article were resolved by the decision of a third reviewer. If the disagreement persisted, the final decision was made by consensus among the three reviewers.

Unavailable articles were requested from the librarians of the State University of Campinas and submitted to full-text analysis.

The following data were extracted from the articles: author; years of publication; country; sample size (n); age (in years); prevalence of weight dissatisfaction in boys, girls, and both sexes; test/measure of epidemiological association; and instrument (scale or questionnaire).

## RESULTS

The 34 articles included in this review were published between 1997 and 2020 (14 were published between 2008 and 2013). The countries that most investigated weight dissatisfaction among adolescents were Brazil (eight studies), the United States (five studies), and Korea (three studies). The sample size ranged from <150 to >103,000 individuals. The age group most analyzed was 12-18 years (four studies).

The prevalence of weight dissatisfaction among adolescents aged 10-19 years ranged from 18.0 to 56.6% in both sexes, from 10.8 to 82.5% among boys, and from 19.2 to 83.8% among girls. These data are given in Tables 1, 2, 3.

**Table 1:** Prevalence of dissatisfaction with body weight among male and female adolescents.

Author	Year	Country^*^	Total (n)	Age (years)	Dissatisfied (%)	Test/association measure^****^	Instrument^*****^
Savage et al.^28^	1997	AU and US	US=820; AU=219	US=13; AU=13	US=43.5; AU=31.1	Chi-square	Q
Wang et al.^18^	2009	US^a^	448	11 (SD=1.0)^**^	24.2	Chi-square	Q
Yeung^38^	2010	Hong Kong	806	11-18	32.0	Chi-square	S
Larson et al^.^ ^15^	2012	New Zealand	8,704	12-18	18.0 (16.5-19.3^)***^	OR	S
Fredrickson et al.^39^	2013	Australia	2,954	11-18	46.8	Chi-square	S
Christofaro et al.^1^	2015	Brazil^b^	2,288	10-17	56.6	Chi-square	Q
Sampasa-Kanyinga et al.^19^	2016	Canada^c^	4,468	11-19	35.5	Pearson chi-square	Q
Xu et al^.^ ^40^	2018	US	2,296	12-17	55.8	OR	Q
Jalali-Farahani et al.^46^	2019	Iran^d^	575	12-18	34.1	Chi-square	Q
Moehlecke et al.^41^	2019	Brazil^e^	71,740	12-17	45.0 (44.0-46.0)^***^	Prevalence ratio	Q
Martini et al.^20^	2020	Brazil^f^	822	10-19	48.8	Rao-Scott chi-square	Q

^*^AU: Australia, US: United States; ^a^Chicago; ^b^Londrina-Paraná; ^c^Ontario; ^d^Isfahan; ^e^273 municipalities; ^f^Campinas-São Paulo; ^**^SD: standard deviation; ^***^95%CI: 95% confidence interval; ^****^measure of epidemiological association; OR: *Odds Ratio*; ^*****^Q: questionnaire, S: scale.

**Table 2:** Prevalence of dissatisfaction with body weight among male adolescents.

Author	Year	Country^*^	Total (n)	Age (years)	Dissatisfied (%)	Test/association measure^****^	Instrument^*****^
Savage et al.^28^	1997	AU and US	US=229; AU=147	US=13; AU=13	US=33.2; AU=24.7	Chi-square	Q
Tomori et al.^25^	2000	Slovenia	2,193	14-19	32.8	Chi-square	Q
Mikkilä et al.^42^	2003	Finland	29,718	14-16	34.0	OR	Q
Park et al.^17^	2003	Korea^a^	1,724	11-18	39.9	Chi-square	Q
Page et al.^21^	2005	Thailand^b^	816	16 (SD=1.3)^**^	54.4	Chi-square	S
Meland et al.^43^	2007	Norway	2,547	11, 13, 15	21.0	OR/RR	Q
Al Sabbah et al.^44^	2008	Palestine	6,867	12-18	29.9	OR	Q
Chen et al.^22^	2008	Taiwan^c^	452	12-16	82.3	OR	S
Sano et al.^24^	2008	Japan and Vietnam	JP=191; VN=352	12-15	JP=51.3; VN=54.8	Chi-square	S
Al Sabbah et al.^3^	2009	24 countries	103,982	11, 13, 15		OR	Q
Kanaan et al.^45^	2010	Lebanon^e^	677	13-19	23.0	OR	Q
Khor et al.^26^	2009	Peninsular Malaysia^f^	1,037	11-15	63.3	Chi-square	S
Kim et al^.^ ^47^	2009	Korea^g^	211	10-13	34.4	Chi-square	Q
Wang et al.^18^	2009	The United States^h^	196	11 (SD=1.0)^**^	15.9	Chi-square	Q
Duca et al.^48^	2010	Brazil^i^	2,044	15-19	42.8	OR	Q
Yeung^38^	2010	Hong Kong	328	11-18	24.0	Chi-square	S
Larson et al.^15^	2012	New Zealand	4,667	12-18	10.8 (9.8-11.8)^***^	OR	S
Suliburska et al.^27^	2012	Poland^j^	300	18	35.0	Chi-square	Q
Wood et al.^16^	2012	New Zealand	4,664	12-18	39.3	OR	S
Fredrickson et al.^39^	2013	Australia	1,660	11-18	36.6	Chi-square	S
Costa et al.^49^	2016	Brazil^k^	80	10-19	82.5	Chi-square	S
Sampasa-Kanyinga et al^.^ ^19^	2016	Canada^l^	2,014	11-19	31.2	Chi-square	Q
Livazović et al.^50^	2017	Croatia^m^	37	15-18	45.9	Chi-square	S
Ra et al.^51^	2017	Korea^n^	17,369	12-15	32.3	Chi-square	Q
Ren et al.^23^	2018	China^o^	1,927	11-16	27.9	Chi-square	Q
Xu et al.^40^	2018	The United States	1,105	12-17	54.0	OR	Q
Jalali-Farahani et al.^46^	2019	Iran^p^	275	12-18	28.8	Chi-square	Q
Moehlecke et al.^41^	2019	Brazil^q^	32,283	12-17	36.4 (35.1-37.7)^***^	Prevalence ratio	Q

^*^AU: Australia, US: United States, JP: Japan, VN: Vietnam; ^a^Seoul; ^b^Chiang Mai; ^c^Taipei; ^d^Flanders; ^e^Beirute; ^f^Penangand Kedah; ^g^Seoul; ^h^Chicago; ^i^Santa Catarina; ^j^Wielkopolska; ^k^Campo Grande; ^l^Ontario; ^m^Osijek; ^n^17 provinces; ^o^Beijing, Shenyang, Zhengzhou, Chongqing, and Guangzhou; ^p^Isfahan; ^q^273 municipalities; ^**^SD: standard deviation; ^***^95%CI: 95% confidence interval; ^****^measure of epidemiological association; OR: *Odds Ratio*; RR: relative risk; ^*****^Q: questionnaire; S: scale.

**Table 3:** Prevalence of dissatisfaction with body weight among female adolescents.

Author	Year	Country^*^	Total (n)	Age (in years)	Dissatisfied (%)	Test/association measure^****^	Instrument^*****^
Savage et al.^28^	1997	AU and US	US=592; AU=73	US=13; AU=13	US=47.4; AU=47.5	Chi-square	Q
Tomori et al.^25^	2000	Slovenia	2,507	14-19	65.5	Chi-square	Q
Sherwood et al^.^ ^54^	2001	US^a^	234	10-13	19.2	t-test	S
Mikkilä et al.^42^	2003	Finland	30,534	14-16	46.0	OR	Q
Park et al.^17^	2003	Korea^b^	1,658	11-18	54.5	Chi-square	Q
Page et al.^21^	2005	Thailand^c^	1,646	16 (SD=1.3)^**^	74.5	Chi-square	S
Meland et al.^43^	2007	Norway	2,479	11, 13, 15	56.0	OR/RR	Q
Al Sabbah et al.^44^	2008	Palestine	8,165	12-18	33.9	OR	Q
Chen et al.^22^	2008	Taiwan^d^	431	12-16	83.8	OR	S
Sano et al.^24^	2008	Japan and Vietnam	JP=174; VN=362	12-15	JP=83.3; VN=57.5	Chi-square	S
Al Sabbah et al.^3^	2009	24 countries	103,982	11, 13, 15		OR	Q
Kanaan et al.^45^	2010	Lebanon^f^	617	13-19	31.0	OR	Q
Khor et al.^26^	2009	Peninsular Malaysia^g^	1,006	11-15	72.7	Chi-square	S
Kim et al.^47^	2009	Korea^h^	194	10-13	32.1	Chi-square	Q
Wang et al.^18^	2009	US^i^	252	11 (SD=1.0)^**^	30.6	Chi-square	Q
Duca et al.^48^	2010	Brazil^j^	2,984	15-19	59.7	OR	Q
Yeung^38^	2010	Hong Kong	478	11-18	37.0	Chi-square	S
Larson et al.^15^	2012	New Zealand	4,037	12-18	26.2 (24.7-27.7)^***^	OR	S
Suliburska et al.^27^	2012	Poland^k^	300	18	65.0	Chi-square	Q
Wood et al.^16^	2012	New Zealand	4,034	12-18	61.4	OR	S
Fredrickson et al.^39^	2013	AU	1,294	11-18	59.8	Chi-square	S
Leme et al.^29^	2013	Brazil^l^	63	13-19	52.4	Chi-square	S
Palma et al.^52^	2013	Brazil^m^	2,149	14-18	42.4	OR	Q
Marques et al.^53^	2014	Brazil^m^	1,082	14-15	48.3	Prevalence ratio	Q
Costa et al.^49^	2016	Brazil^n^	133	10-19	78.9	Chi-square	S
Sampasa-Kanyinga et al.^19^	2016	Canada^o^	2,454	11-19	40.0	Chi-square	Q
Livazović et al.^50^	2017	Croatia^p^	111	15-18	61.2	Chi-square	S
Ra et al.^51^	2017	Korea^q^	16,005	12-15	45.6	Chi-square	Q
Ren et al.^23^	2018	China^r^	1,914	11-16	41.2	Chi-square	Q
Xu et al.^40^	2018	US	1,191	12-17	57.4	OR	Q
Jalali-Farahani et al.^46^	2019	Iran^s^	300	12-18	39.0	Chi-square	Q
Moehlecke et al.^41^	2019	Brazil^t^	39,457	12-17	53.8 (52.2-55.3)^***^	Prevalence ratio	Q

^*^AU: Australia, US: United States, JP: Japan, N: Vietnam; ^a^Minnesota and Wisconsin; ^b^Seoul; ^c^Chiang Mai; ^d^Taipei; ^e^Flanders; ^f^Beirute; ^g^Penangand Kedah; ^h^Seoul; ^i^Chicago; ^j^Santa Catarina; ^k^Wielkopolska; ^l^São Paulo; ^m^Rio de Janeiro; ^n^Campo Grande; ^o^Ontario; ^p^Osijek; ^q^17 provinces; ^r^Beijing, Shenyang, Zhengzhou, Chongqing, and Guangzhou; ^s^Isfahan; ^t^273 municipalities; ^**^SD: standard deviation; ^***^95%CI: 95% confidence interval; ^****^measure of epidemiological association; OR: odds ratio; RR: relative risk; ^*****^Q: questionnaire; S: scale.

Al Sabbah et al.^3^ examined 24 countries and regions in Europe, Canada, and the United States and found a lower prevalence in the Netherlands (34.1%) and Russia (36.0%) and a higher prevalence in the Czech Republic (61.8%) and Slovenia (56.8%) in female adolescents. In male adolescents, the lower prevalence was found in Ukraine (14.1%) and Russia again (15.4%) and the higher prevalence in Italy (39.9%) and the United States (37.7%).

Although the prevalence of weight dissatisfaction was high in the majority of countries, Larson et al.^15^ identified lower rates in New Zealand: 18.0% (95%CI 16.5-19.3) in both sexes, 26.2% (95%CI 24.7-27.7) among girls, and 10.8% (95%CI 9.8-11.8) among boys. However, another study also conducted in New Zealand found rates more than twofold higher than those described by Larson et al.,^15^ reporting that 61.4% of girls and 39.3% of boys were dissatisfied with their weight.^16^

When analyzing the Brazilian studies, the prevalence of body weight dissatisfaction in both sexes was 56.6% in Londrina, PR, 48.8% in Campinas, SP, and 45.0% (95%CI 44.0-46.0) in adolescents of 273 Brazilian municipalities with more than 100,000 inhabitants. While among boys, the prevalence of weight dissatisfaction was 82.5% in Campo Grande, MS, 42.8% at public schools in Santa Catarina, and 36.4% (95%CI 35.1-37.7) in boys of 273 Brazilian municipalities, among girls, the prevalence of weight dissatisfaction was 78.9% in Campo Grande, MS, 59.7% in Santa Catarina, 53.8% (95%CI 52.2-55.3) in girls of 273 Brazilian municipalities, 52.4% in São Paulo, SP, and 48.3 and 42.4% in Rio de Janeiro, RJ.

Statistically significant differences in weight dissatisfaction were found in the comparison between boys and girls (p<0.01;^17^ p=0.001;^18^ p<0.001),^3^ ^,^ ^19^ ^,^ ^20^ ^,^ ^21^ ^,^ ^22^ ^,^ ^23^ ^,^ ^24^ ^,^ ^25^ ^,^ ^26^ ^,^ ^27^ in the comparison of American and Australian samples (p<0.05),^28^ in the comparison of American and Australian boys (p<0.05),^28^ and in the comparison of dissatisfaction/satisfaction/indifference with weight among girls (p<0.001).^29^

## DISCUSSION

Differences in the prevalence of weight dissatisfaction of adolescents among countries and capitals/states of Brazil demonstrate the particularities of each culture as well as social and economic factors.^24^ ^,^ ^14^

Society values thinness in the female sex and strength in the male sex,^24^ which often makes boys more concerned with muscle size than weight,^30^ whereas girls are more dissatisfied with their weight, as found in the majority of studies evaluated. Adolescents strive to meet these standards of beauty and the expectations of the society in which they live.^31^ Data from the National Adolescent School-Based Health Survey (PeNSE, 2015) revealed that the majority (84.1%) of students aged 13-17 years considered their body image as important or very important (86.2% of girls vs. 81.9% of boys), 18.3% of students considered themselves fat or very fat (21.8% of girls vs. 14.6% of boys), and 25.6% wanted to lose weight (30.3% of girls vs. 20.5% of boys). In terms of gaining weight/muscle mass, this desire was observed in 16.3% of students (15.4% of girls vs. 17.2% of boys).^32^

Exposure to traditional media (i.e., television, cinema, and magazines) and social media can lead to body weight dissatisfaction among adolescents, making them more susceptible to the internalization of an unrealistic, idealized stereotype. Social media enable users to create their own content and publish photographs of themselves, giving them the opportunity to become more attractive as well as compete with and compare themselves to others.^19^ ^,^ ^33^ The use of social media for more than 2 h/day is associated with an increased desire to become thin among girls, whereas the use of social media for 2h/day or less is associated with a lower risk of self-perception of overweight in boys.^19^

The involvement of parents in the physical and emotional development, and the active participation in education are fundamental in the prevention of weight dissatisfaction,^3^ ^,^ ^34^ promotion, and encouragement of the positive formation of their children’s body image.^34^ The school environment can also contribute to the development of skills in adolescents to cope with self-esteem and body dissatisfaction, such as a reduction in bullying through the implementation of programs that incorporate teacher training and student activities, addressing weight-related teasing.^35^ Thus, parents and teachers play essential roles in the construction of a healthy body image and the relation to adequate weight and can act preventively even before the arrival of adolescence.

Adolescence is a transition phase between childhood and adulthood marked by physical, psychological, and social changes, including changes in body size and shape. Moreover, puberty, which is the result of sexual and reproductive development, further accentuates concerns with one’s body image and weight,^14^ which makes this population more easily influenced by sociocultural model of thinness as the best body shape.^34^ ^,^ ^36^ This value should be changed as it can contribute to the development of unhealthy habits.^34^ Therefore, the issue of weight dissatisfaction merits attention, as it can interfere with the growth and development of adolescents and lead to behaviors that place one’s health at risk.

Considering the observed changes in the epidemiological and nutritional patterns of populations, WHO recommends that the governments elaborate and update the dietary guidelines for the population, using a language accessible to everyone, in order to optimize the adoption of healthier food choices. Thus, the Dietary Guidelines for the Brazilian Population aim to promote healthy eating habits based on the consumption of fresh or minimally processed foods. In Brazil, as in most countries, the prevalence of excess weight, as well as chronic diseases related to excessive food consumption and ultraprocessed food is increasing rapidly, and many of these problems are affecting young adults, adolescents, and children. The involvement of adolescents in the purchase and preparation of food allows them to learn more about where the food comes from, how they are produced, and new possibilities of preparations, which is an excellent opportunity for the adolescents to incorporate good habits and value the importance of regular and balanced meals and realization of healthy environments. In addition, more than two-thirds of commercials about food shown on television are ultraprocessed foods targeted directly at children and adolescents, who are forming eating habits that could be lifelong.^37^

It is worth mentioning that the lack of standardization due to the use of different methods (i.e., questionnaires and scales) in the studies made it necessary to unify the data to extract comparable information on weight dissatisfaction. Numerous studies presented specific data to categorize body weight dissatisfaction^1^ ^,^ ^3^ ^,^ ^17^ ^,^ ^19^ ^,^ ^24^ ^,^ ^28^ ^,^ ^29^ ^,^ ^38^ ^,^ ^39^ ^,^ ^40^ ^,^ ^41^ ^,^ ^42^ ^,^ ^43^ ^,^ ^44^ ^,^ ^45^ and others offered the options dissatisfied/satisfied/neither satisfied or dissatisfied.^23^ ^,^ ^46^ ^,^ ^47^ Other studies evaluated weight dissatisfaction using categories, such as “wants to gain weight/lose weight” (some articles stipulated cutoff points, such as >10% of current weight) and satisfaction.^20^ ^,^ ^21^ ^,^ ^25^ ^,^ ^26^ ^,^ ^27^ ^,^ ^48^ ^,^ ^49^ ^,^ ^50^ ^,^ ^51^ ^,^ ^52^ ^,^ ^53^ Some articles show weight dissatisfaction based on statements that expressed unhappiness or happiness with one’s own weight^15^ ^,^ ^16^ ^,^ ^18^ ^,^ ^54^ and the discrepancy between the self-evaluation of current weight and idealized weight.^22^

Due to the complexity of this issue, the present study did not extract data on the associations between weight and weight dissatisfaction, nutritional status, and self-evaluation. As the purpose of the present study was to evaluate weight dissatisfaction according to sex, it was not possible to identify whether the adolescents with lower rates of dissatisfaction were in the ideal weight range or whether or not self-evaluated weight corresponded to actual weight.

The articles selected had a cross-sectional design, which does not enable drawing conclusions on the direction of causality between the variables; therefore, this study is not representative, in its entirety, in the scientific literature, as the purpose of the study was to analyze the prevalence of weight dissatisfaction.

Based on the findings of this systematic review, the prevalence of body weight dissatisfaction is high among adolescents, especially girls. Such information can contribute to the planning of health and education programs directed at adolescents and weight (dis)satisfaction, which are necessary to the prevention/reduction of emotional, physical, and mental problems. Besides that, the formulation and execution of public policies, and intersectional and multidisciplinary actions such as in health services, school and family environments are essential to teach and protect mainly children and adolescents to be critical from exposure of food advertising and marketing, support and encourage healthy eating practices individually and collectively, and prevent and promote the health and the food and nutrition security in this population.
